# Modulation of Rhythmic Activity in Mammalian Spinal Networks Is Dependent on Excitability State

**DOI:** 10.1523/ENEURO.0368-16.2017

**Published:** 2017-01-27

**Authors:** Simon A. Sharples, Patrick J. Whelan

**Affiliations:** 1 Hotchkiss Brain Institute, University of Calgary, Calgary, Alberta T2N 4N1, Canada; 2 Department of Comparative Biology and Experimental Medicine, University of Calgary, Calgary, AB T2N 4N1, Canada

**Keywords:** CPG, locomotion, network, spinal cord

## Abstract

Neuromodulators play an important role in activating rhythmically active motor networks; however, what remains unclear are the network interactions whereby neuromodulators recruit spinal motor networks to produce rhythmic activity. Evidence from invertebrate systems has demonstrated that the effect of neuromodulators depends on the pre-existing state of the network. We explored how network excitation state affects the ability of dopamine to evoke rhythmic locomotor activity in the neonatal mouse isolated spinal cord. We found that dopamine can evoke unique patterns of motor activity that are dependent on the excitability state of motor networks. Different patterns of motor activity ranging from tonic, nonrhythmic activity to multirhythmic, nonlocomotor activity to locomotor activity were produced by altering global motor network excitability through manipulations of the extracellular potassium and bath NMDA concentration. A similar effect was observed when network excitation was manipulated during an unstable multirhythm evoked by a low concentration (15 µm) of 5-HT, suggesting that our results are not neuromodulator specific. Our data show in vertebrate systems that modulation is a two-way street and that modulatory actions are largely influenced by the network state. The level of network excitation can account for variability between preparations and is an additional factor to be considered when circuit elements are removed from the network.

## Visual Abstract

**Figure:**
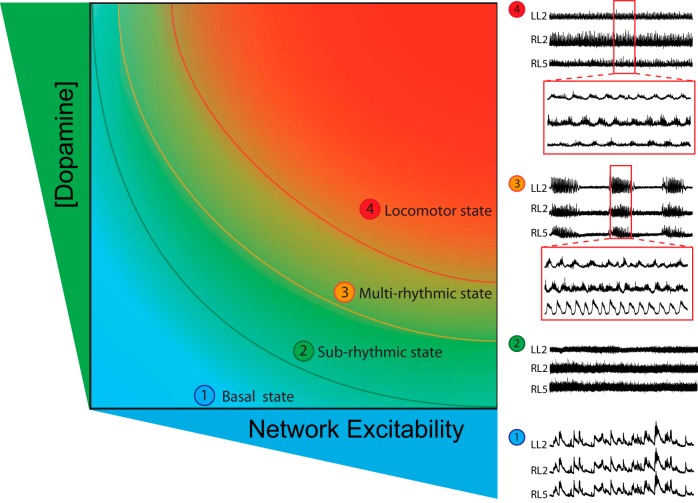

## Significance Statement

We show that, as in the invertebrate systems, the action of monoamine modulators on rhythmic motor networks of the mammalian spinal cord is state dependent. Our work shows that neuromodulation in the spinal cord is fundamentally linked to the excitability state of the network. These findings have broad significance on mammalian network function since variations in network excitation can account for (1) diversity of neuromodulator function, which (2) is an additional factor that must be considered when circuit elements are removed from a network to infer network function and (3) can account for the variability often found between experimental preparations.

## Introduction

Rhythmic motor behaviors mediate a number of functions essential to survival, including breathing (Smith et al., 1991; Baghdadwala et al., 2016), feeding (Blitz and Nusbaum, 2012; Stadele et al., 2015), and locomotion (Gordon and Whelan, 2006; Grillner et al., 2008; Sillar et al., 2014; Kiehn, 2016). In vertebrates, neuronal networks within the spinal cord control rhythmic movements of the limbs and axial musculature to produce various forms of locomotion (Hultborn and Nielsen, 2007; Roberts et al., 2012; Kimura et al., 2013; Kiehn, 2016). While these networks have the capacity to function in isolation, normally they are modulated by inputs from the brain (Miles and Sillar, 2011; Koblinger et al., 2014; Sharples et al., 2015) and brainstem (van den Pol, 1999; Hentall et al., 2003; Jordan et al., 2008; Bouvier et al., 2015), and by sensory feedback from the limbs (Whelan et al., 1995). Collectively, these inputs endow these networks with the flexibility to produce diverse patterns of rhythmicity to suit the task at hand (Jordan et al., 2008; Sillar et al., 2014; Marder et al., 2015). Although evoked rhythms in the spinal cord have been characterized by others including our group, and diversity reported, we are not aware of this diversity being explained in terms of a modulatory-excitation state space (Cowley and Schmidt, 1997; Pearson et al., 2003; Madriaga et al., 2004; Barrière et al., 2005; Christie and Whelan, 2005; Gordon and Whelan, 2006; Puhl and Mesce, 2008; Humphreys and Whelan, 2012; Gozal et al., 2014; Heckman and Johnson, 2014).

The importance of excitation state is evident from examples provided in the small circuits of the stomatogastric nervous system whereby different motor patterns can be modeled as a function of balanced synaptic weights as well as intrinsic properties (Gutierrez et al., 2013; Gutierrez and Marder, 2014). The range of motor patterns generated as a function of varying circuit properties has been termed the circuit state or parameter space. Based on this, it has been proposed that the modulation of circuit output is state dependent and that the influence of a neuromodulator is linked to the relative position of the circuit within its parameter space. Several lines of evidence demonstrates that specific motor patterns can also be achieved through multiple or degenerate mechanisms (Prinz et al., 2004; Gutierrez et al., 2013; Marder et al., 2014).

We show that, as in the invertebrate systems (Bargmann, 2012; Marder et al., 2014), the action of monoamine modulators on rhythmic motor networks of the mammalian spinal cord is state dependent. We explore how network excitation state influences the modulatory effect of dopamine on rhythmically active motor networks in the isolated spinal cord of neonatal mice. This model is advantageous since elements of the circuit have been genetically defined, and the motor output has been well characterized by several laboratories and is starting to be mathematically modeled. Broadly speaking, our data suggest that it is important to consider modulation as a two-way street; modulatory actions are largely influenced by the network state. Our results have functional implications in pathological instances such as in multiple sclerosis, spinal cord injury, and stroke, where spinal cord excitability and stepping performance are compromised. Furthermore, the irregular rhythmic movements we report here may be manifest in neonatal rodents, while spinal networks are maturing and descending projections are developing. At this time, the neonate must still make movements to move toward the mother. These movements tend to be erratic compared with the full-fledged stepping that occurs around postnatal day 9 (P9) to P10 when complete weight support is realized. Our data also function to provide a reference that investigators can use to interpret variability in rhythmic patterns observed using isolated spinal cord preparations. A portion of these results has been published in abstract form (Sharples and Whelan, 2015).

## Materials and Methods

### Ethical approval and animals

Experiments were performed on male and female neonatal C57BL/6 mice that were 0-4 days old (P0–P3; *N* = 82). All procedures used were approved by the University of Calgary Health Sciences Animal Care Committee.

### Tissue preparation

Animals were anesthetized by cooling, decapitated, and eviscerated to expose the vertebral column. The remaining tissue was placed ventral side up in a dissection chamber filled with room temperature carbogenated (95% O_2_, 5% CO_2_) artificial CSF (aCSF; in mm: 128 NaCl, 4 KCl, 1.5 CaCl_2_, 1 MgSO_4_, 0.5 Na_2_HPO_4_, 21 NaHCO_3_, and 30 d-glucose), and spinal cords were exposed via a ventral laminectomy and the dorsal and ventral roots cut. The spinal cord was removed, transferred to a recording chamber, ventral side up, with recirculating carbogenated aCSF at a flow rate of 20 ml/min and gradually heated from room temperature to 27ºC. This temperature has been used historically by our group and can control for temperature variability when experiments are conducted at room temperature. The spinal cord was allowed another 45–60 min to stabilize in the recording chamber.

### Electrophysiological recordings

Neurograms obtained from ventral roots of the lumbar 2 (L2) segments and the L5 were amplified (1000 times; EX-4, Dagan), bandpass filtered (0.1–1 kHz), digitized (Digidata 1440, Molecular Devices), acquired (2.5 kHz) using Clampex software (Molecular Devices), and saved for off-line analysis. Recorded motor activity was analyzed using custom-written MATLAB scripts, Spike2 (Cambridge Electronics) and Spinal Core (a gift from Professor A. Lev-Tov, Hebrew University of Jerusalem, Jerusalem, Israel; Mor and Lev-Tov, 2007).

### Pharmacology

Patterns of rhythmic motor activity were evoked by bath application of dopamine hydrochloride (Sigma-Aldrich) or serotonin [5-hydroxytryptamine (5-HT)] hydrochloride (Sigma-Aldrich). Spinal motor network excitability was manipulated pharmacologically by altering the concentration of KCl (VWR) in the bath, applying concentrations of NMDA (2-6 µm; Sigma-Aldrich) that were subthreshold to those that are capable of evoking locomotor patterns of activity in our hands (10-12 µm) and concentrations of MgSO_4_ (1.0-2.5 mm; VWR) to reduce but not suppress all network activity. The NMDA receptor antagonist aminophosphonovalerate (APV) was also bath applied at concentrations capable of reducing but not suppressing spontaneous activity (5 µm; Tocris Bioscience).

### Data analysis

**Figure 1.**
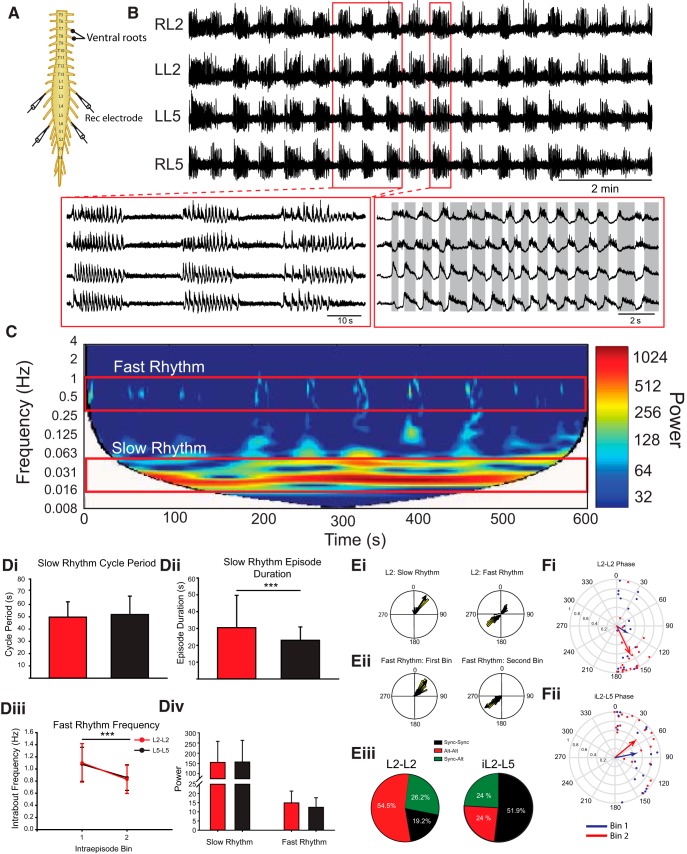
Dopamine evokes multirhythmic patterns of motor activity. ***A***, Neurograms recorded from the left and right ventral roots of the L2 and L5 isolated thoracolumbar spinal cord. ***B***, Dopamine evokes a multirhythmic pattern of motor activity that is composed of two rhythms: a slow, synchronous rhythm with a superimposed fast rhythm. ***C***, These fast and slow rhythms (red boxes) are apparent when the frequency power is represented over time as a spectrogram with frequency on the left *y*-axis, time on the *x*-axis, and warmer colors representing higher frequency power. ***Di***, ***Dii***, ***Div***, The slow rhythm features including cycle period (***Di***), episode duration (***Dii***), and power (***Div***) did not differ between L2 (red bars) and L5 (black bars) segments. ***Diii***, ***Div***, The frequency of the fast rhythm occurring within bouts (***Diii***) decreases over the course of the bout (***Div***) with no difference in power between L2 and L5 (***Div***). Data are displayed as the mean ± SD. ***Ei***, Example phase plots from a single bout of rhythmic activity recorded from the L2 segment illustrate that the bouts are synchronous, with the fast superimposed rhythm exhibiting a biphasic phase distribution. ***Eii***, Separating the fast rhythm into two bins illustrates that the fast bursts start off synchronous and end alternating. ***Eiii***, The pie charts represent the distribution of the possible patterns over the course of a bout recorded between the left and right L2 and ipsilateral L2–L5 ventral roots. ***Fi***, Reflects the mean phase of all bouts. ***Fii***, The predominantly synchronous bursting pattern in ipsilateral ventral root pairs is represented in the mean phase plot. The phases in circular plots are reported in degrees with the length of arrows representing mean vector length (*r*) and angle.

Patterns of rhythmic motor activity were analyzed by performing a Morlet cross-wavelet analysis on ventral root recordings made from left and right L2 and L5 spinal segments and ipsilateral L2–L5 pairs (Mor and Lev-Tov, 2007). Autowavelet spectral analysis was conducted on single ventral root recordings for dopamine dose-response experiments. Spectrograms are displayed with a white V-shaped cone of influence. Frequency power spectrograms were constructed and data were analyzed by selecting regions of interest within the cone of influence. The spectrograms illustrate two distinct rhythms evoked by dopamine at high concentrations, a slow rhythm of 0.01–0.04 Hz, and a fast rhythm at 0.8–1.2 Hz (Fig. 1*B*). Regions of interest were selected within these frequency ranges and analyzed over the time course of each experiment. Parameters measured within each region of interest include frequency and power. Circular statistics were also examined to explore aspects of the pattern of bursting between neurograms recorded from root pairs (i.e., alternating, 180°; or synchronous, 0°/360°) and include the phase relationship (vector angle) between bursts in the left and right or L2–L5 roots and phase vector length (*r*). Data analyzed within regions of interest over the course of each experiment were segmented into 30 s bins and further averaged over 5 min intervals for statistical analysis. All analyses conducted on rhythmic motor activity were conducted using the data analysis tools available in Spinal Core (Mor and Lev-Tov, 2007).

### Statistics

Repeated-measures ANOVAs were conducted for experiments that involved manipulation of the network excitation state after the addition of dopamine (i.e., KCl or NMDA). Tukey *post hoc* analyses were conducted when significant main effects were detected with *p* < 0.05. One-way ANOVAs were performed for excitability reduction experiments. *Post hoc* analyses were performed comparing the frequency power for each experiment to frequency power of rhythms evoked by 50 µm dopamine alone in previous experiments. Nonparametric Friedman repeated-measures ANOVA or Mann–Whitney rank sum tests were conducted when assumptions of normality or equal variance were violated using a Shapiro–Wilk test and a Brown–Forsythe test, respectively. Power analyses could not be conducted on nonparametric statistics.

## Results

### Dopamine evokes multiple patterns of rhythmic motor activity

We first examined the rhythmic activity evoked only by dopamine, a neuromodulator often used to modulate locomotor activity (Whelan et al., 2000; Bonnot et al., 2002; Barrière et al., 2004; Humphreys and Whelan, 2012; Sharples et al., 2015; Picton et al., 2016). The rhythm that emerged following bath application of 50 µm dopamine does not resemble a fictive locomotor pattern, which is typically characterized by continuous and alternating bursts between left and right sides within segments and alternation between ipsilateral L2 and L5 bursts. Instead, the rhythm evoked by dopamine consists of a slow and a fast component. The slow episodes occurred synchronously across left and right ventral roots in the L2 (phase: 20.5°; *r* = 0.89; *p* < 0.0001) and L5 (phase: 16.4°; *r* = 0.87; *p* < 0.0001; Fig. 1*A*,*Ei*) and between ipsilateral ventral root bursts in the L2 and L5 (phase: 14.0°; *r* = 0.91; *p* < 0.0001). The bouts had a mean cycle period of 50 ± 15 s (Fig. 1*Di*) with the onset phase locked across L2 and L5 bursts. Episode durations are significantly longer in L2 segments compared to L5 possibly reflecting a rostrocaudal gradient of excitability for dopamine (Christie and Whelan, 2005; Fig. 1*Dii*; L2, 30.5 ± 19 s; L5, 23.0 ± 7.9 s; Wilcoxon signed rank test: W = −160, T+ = 25, T− = −185, *z* = −2.99, *p* = 0.002). The slow rhythm can be observed in the high power region within the frequency-power spectrogram around 0.02 Hz (Fig. 1*C*).

The slow-depolarizing episodes activated a faster rhythm that was superimposed on the episodes and is illustrated in the fast rhythm band in Figure 1*B*. In both the L2s and the L5s, the fast rhythm slows from 1.1 ± 0.3 to 0.83 ± 0.2 Hz over the course of the episode (Fig. 1*Diii*; L2: *t*_(18)_ = 4.9, *p* < 0.00006; L5: *t*_(18)_ = 4.9, *p* = 0.00005) with no change in rhythm power over the course of an episode and no difference in power between L2 and L5 segments (Fig. 1*Div*). We found instances when the pattern of bursts between ventral root neurograms of left and right L2, left and right L5, or ipsilateral L2–L5 pairs within the fast rhythm start off synchronous and switched to an alternating pattern toward the end of the episode (Fig. 1*Eii*). There are also episodes where the fast rhythm is either alternating or synchronous between root pairs for the duration of the episode. Of note, the pattern never started off alternating and changed to synchrony. Examination of the fast rhythm in all episodes in the left and right L2 neurograms revealed that the predominant pattern consists of a fast rhythm that starts off alternating and finishes alternating (54.5% of episodes). This is in contrast to the predominant pattern of bursting between ipsilateral L2 (iL2) and iL5 left–right (L–R) L5, which started off as synchronous and ended as synchronous (iL2–L5, 51.8% of bursts; L–R L5, 85.8% of episodes; Fig. 1*Eiii*). The predominant burst pattern within each ventral root pair is reflected in the mean vector plots when the data from all episodes are averaged as the average pattern is alternating within the L2s (Fig. 1*Fi*; phase: 154.4°; *r* = 0.65; Rayleigh test, *p* = 0.0001), synchronous in the L5s (phase: 20.3°; *r* = 0.72; Rayleigh test, *p* = 0.00002), and biased toward synchrony in the ipsilateral L2–L5s (Fig. 1*Fii*; phase: 78.6°; *r* = 0.45; *p* = 0.02).

**Figure 2.**
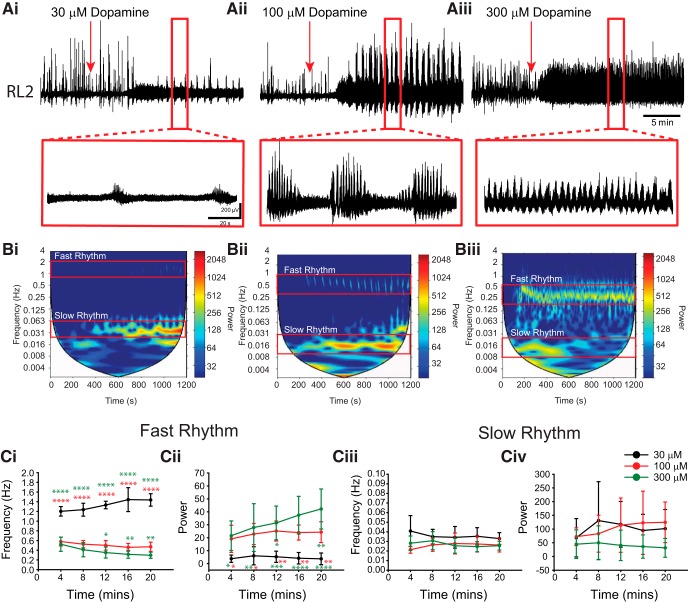
High concentrations of dopamine evoke rhythmicity. ***Ai–Aiii***, Neurograms from single L2 ventral roots from individual experiments with dopamine (DA) bath applied (red arrows) at 30 µm (***Ai***), 100 µm (***Aii***), and 300 µm (***Aiii***) to naive preparations evoke rhythmic motor activity. ***Bi–Biii***, The fast and slow rhythms are illustrated in the autowavelet frequency power spectrograms over time with frequency on the left *y*-axis, time on the *x*-axis, and warmer colors representing higher power. Rhythm frequency and power of the fast and slow rhythms were analyzed by selecting regions of interest selected within bouts of the fast rhythm and within the range of the slow rhythm. ***Ci***, ***Cii***, The frequency of the fast rhythm slowed down (***Ci***) and power increased (***Cii***) with dopamine concentration. ***Ciii***, ***Civ***, No differences were found in frequency (***Ciii***) or power (***Civ***) of the slow rhythm between dopamine concentrations. Data are presented as the mean ± SD, with asterisks denoting significance (*p < 0.05, **p < 0.01, ***p < 0.001) with Tukey *post hoc* test following a two-way ANOVA between time (5 min bins) and dopamine concentration.

Different patterns of rhythmic motor activity were expressed at different concentrations of dopamine tested in a series of experiments (Fig. 2). Recording from single L2 ventral roots, we determined that 30 µm was the lowest concentration of dopamine capable of evoking rhythmicity in five of eight preparations. A 100 µm concentration evoked robust discontinuous multirhythmic activity (seven of nine preparations) or even weak continuous rhythmicity (two of nine preparations), and a 300 µm concentration evoked robust continuous patterns of rhythmicity (six of eight preparations). In rhythmic preparations, the time from the addition of dopamine until the first bout of rhythmicity decreased as a function of dopamine concentration (30 µm = 562 ± 173 s; 100 µm = 338 ± 70 s; 300 µm = 231 ± 46 s; *p* < 0.001). The frequency of the fast rhythm was significantly higher at 30 µm compared with 100 and 300 µm (Fig. 2*Ci*; *F*_(2,19)_ = 186; *p* < 0.001). The fast rhythm slowed down and became more robust (greater power) as a function of dopamine concentration (Fig. 2*Cii*; *F*_(2,19)_ = 20.33; *p* < 0.001) as the rhythm switched from a multirhythm at 100 µm to a continuous rhythm at 300 µm.

### Dopamine-evoked rhythmicity is dependent on network excitation state

The position of the network within parameter state space has been proposed to affect how neuromodulators influence rhythmic motor network output (Marder et al., 2014) and may contribute to the variability we observed with respect to the diversity in rhythmic patterns evoked at different dopamine concentrations. Conceptually, if a neuromodulator is acting around the transition point for a network, where the pattern would change, a similar change in intrinsic properties of a class of neurons within the network would have a large effect compared with when it operates far from the transition borders. We therefore devised a series of experiments whereby the global excitability of spinal motor networks was manipulated by increasing the concentration of extracellular KCl in the bath or by application of NMDA to nonspecifically excite spinal motor networks. The goal here was to move the spinal network around a state space so that we would abut transition zones.

**Figure 3.**
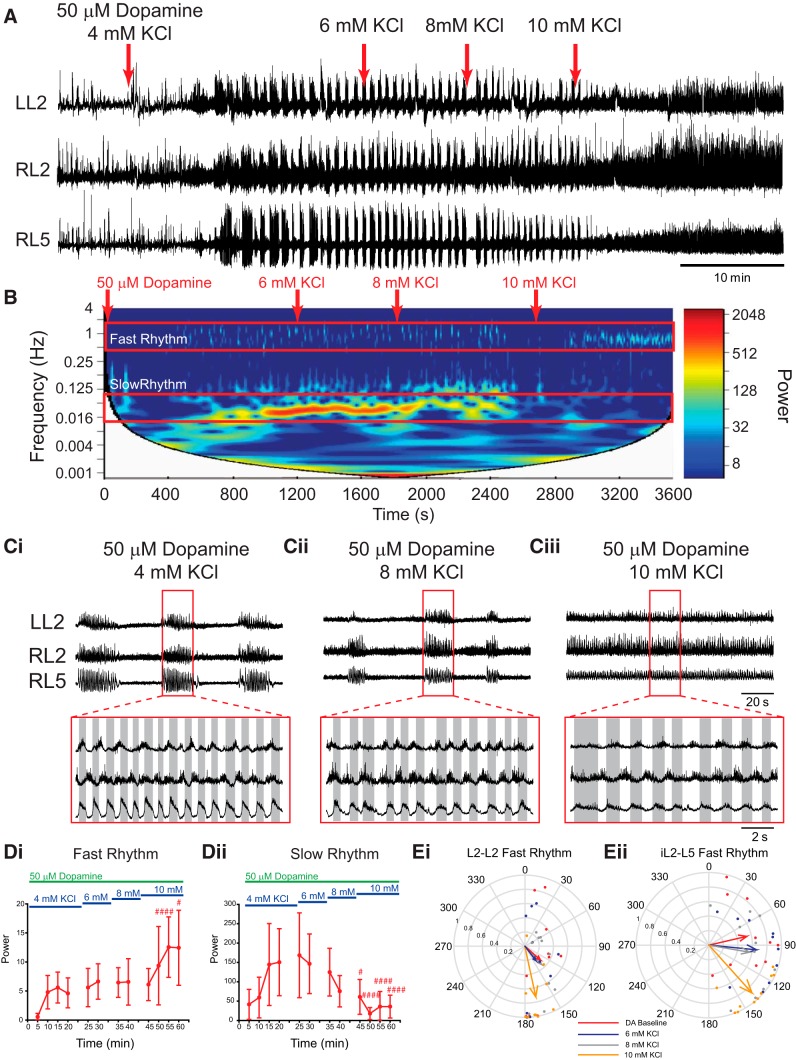
Sequentially boosting network excitation with KCl modulates dopamine-evoked rhythmicity. ***A***, Neurograms recorded from the left and right L2 and right L5 ventral roots show raw data and the effect on rhythmicity. Bath application of 50 µm dopamine evokes a multirhythmic pattern of motor activity that is modulated as network excitation is boosted by sequentially increasing the concentration of KCl in the bath (red arrows). When KCl concentration is increased to 10 mm, the pattern switches from multirhythmic to a single, continuous locomotor-like rhythm. ***B***, The frequency power distribution following dopamine application and subsequent excitability manipulation is illustrated in the cross-wavelet frequency power spectrogram over time with frequency on the left *y*-axis, time on the *x*-axis, and increasing power represented as warmer colors. At 10 mm KCl, the pattern switches from multiple rhythms to a single continuous rhythm. ***Ci–Ciii***, Representative neurograms showing rhythm at baseline (***Ci***), dopamine with 8 mm KCl (***Cii***), and a continuous locomotor-like rhythm expressed with dopamine and 10 mm KCl (***Ciii***). ***D***, Regions of interest were selected around the fast and slow rhythms, and the respective frequency and power for left and right L2 and L5 neurograms were analyzed over time. ***Di***, ***Dii***, Increasing network excitation increased the power of the fast rhythm (***Di***) and decreased the power of the slow rhythm (***Dii***). Data are presented as the mean ± SD, with asterisks denoting a significant difference between the respective point and the rhythm at 20 min following dopamine application (*p < 0.05, **p < 0.01, ***p < 0.001) obtained from Tukey *post hoc* analysis when significant main effects on a repeated-measures ANOVA were found. Nonparametric statistical analysis was performed when assumptions of normality failed, and significance was denoted as follows: #*p* < 0.05, ##*p*<0.01, ###*p* < 0.001. ***Ei***, ***Eii***, The phase between L2 neurograms for the fast rhythm is presented in the circular plots in ***Ei*** and ***Eii*** and illustrate the switch to a locomotor-like pattern at 10 mm KCl as the vector length increases and phase moves toward 180° (alternating) in both the left and right L2s and ipsilateral L2–L5. Each dot represents the average phase for an individual preparation for each respective experimental condition. The length of the arrows represents the mean vector length (*r*) or the robustness of the pattern.

Following bath application of 50 µm dopamine and the generation of a regular multirhythm, the bath KCl concentration was sequentially increased from basal levels (4 mm KCl in the aCSF) in 2 mm increments, and the resultant effects on the rhythm were recorded (Fig. 3; *n* = 10). The duration of the episodes that composed the slow rhythm decreased as KCl was increased, and differences in episode duration in L2 and L5 were no longer apparent at 6 and 8 mm KCl (baseline: L2, 31 ± 26 s; L5, 23.6 ± 9.5 s; 6 mm: L2, 17.7 ± 5 s; L5, 15.0 ± 2.5 s; 8 mm: L2, 12.4 ± 2.4 s; L5, 11.8 ± 2.3 s). Boosting network excitation via KCl decreased the frequency of the fast rhythm (L2: *F*_(11,99)_ = 5.9, *p* < 0.001; L5: *F*_(11, 88)_ = 0.47, *p* = 0.9) and increased the frequency of the slow rhythm (L2: *F*_(11, 99)_ = 3.7, *p* < 0.001) in the L2s. The power of the fast rhythm increased (L2: χ^2^_(11)_ = 53.7, *p* < 0.001; L5: χ^2^_(11)_ = 51.2, *p* < 0.001), and slow rhythm decreased (L2: χ^2^_(11)_ = 63.8, *p* < 0.001; L5: *F*_(10,80)_ = 8.3, *p* < 0.001) in both the L2s and L5s (Fig. 3*Di*,*Dii*) as excitation was increased and is a reflection of the rhythm switching from discontinuous multirhythm to a continuous rhythm. The pattern of the fast rhythm became more locomotor like as excitation was increased, which is indicated by the phase moving toward 180° in the left and right L2s (Fig. 3*Ei*), the ipsilateral L2 and L5 (Fig. 3*Eii*), and the left and right L5s (DA baseline: phase, 13.5°, *r* = 0.75, *p* = 0.004; 10 mm KCl: phase, 145°, *r* = 0.43, *p* = 0.2).

**Figure 4.**
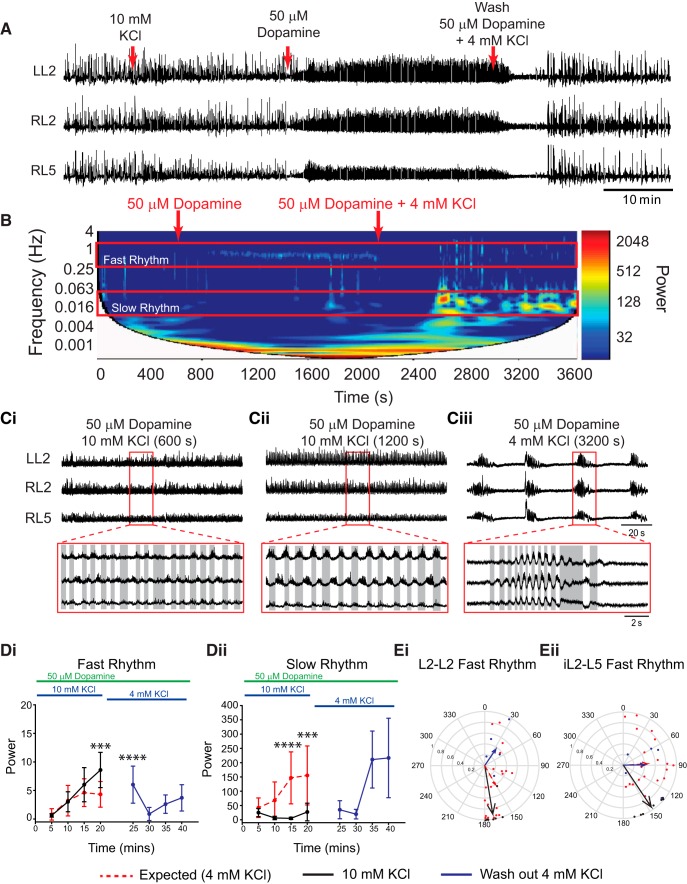
Boosting network excitation with KCl prior to the application of dopamine evokes locomotor-like rhythmicity. ***A***, Neurograms recorded from left and right L2 and right L5 ventral roots illustrate the experimental paradigm and the resultant effect on rhythmicity. KCl concentration was increased to 10 mm to boost network excitation 20 min prior to application of 50 µm dopamine (DA). Subsequent application of DA resulted in the direct expression of a continuous locomotor-like rhythm that returned to a multirhythm when washed out with regular (4 mm KCl) aCSF and 50 µm DA. ***B***, Frequency power spectrogram with frequency on the left *y*-axis, time on the *x*-axis, and increasing power represented as warmer colors. ***Ci***, ***Cii***, Raw data showing zoomed regions represented in ***B*** of 50 µm dopamine plus 10 mm KCl at a longer time point (***Ci***) and following wash in of 4 mm KCl (***Cii***). ***D***, Region-of-interest analysis of fast and slow rhythms within L2 root pairs illustrate significantly higher power of the fast rhythm with 10 mm KCl compared with the expected rhythm power at 4 mm KCl. ***Di***, The slow rhythm showed significantly lower power compared with the expected multirhythm evoked in the 4 mm KCl condition. ***Dii***, The expected power values returned to the same level as the expected condition following a washout with 4 mm KCl (Blue lines). ***Ei***, ***Eii***, Circular plots in ***Ei*** and ***Eii*** illustrate a locomotor-like pattern with vector length increases accompanied by phase angles moving toward 180° (alternating) in both the left and right L2s and ipsilateral L2–L5 at higher KCl concentrations. The length of arrows represents mean vector length (*r*) and angle or robustness of the pattern. Red lines represent mean data (*n* = 20 preparations) when 50 µm DA (aCSF, 4 mm KCl) was applied, and 20 min of baseline data were analyzed. Black lines represent the rhythm evoked by 50 µm DA under enhanced network excitation (aCSF, 10 mm KCl), and blue lines represent the washout condition of DA (aCSF, 4 mm KCl). Each dot in the phase plots represents the average phase for an individual preparation for each respective experimental condition. Data are presented as the mean ± SD. A two-way ANOVA between each excitability condition (DA-evoked rhythm in 4 mm KCl, 10 mm KCl, and washing with 4 mm KCl) and time to examine the effects of manipulating network excitation prior to DA application. When significant main effects of interactions were detected Tukey *post hoc* analysis between time-matched points following DA application were conducted. Asterisks denote significance, as follows: *p < 0.05, **p < 0.01, ***p < 0.001.

These effects were replicated when network excitation was enhanced by the addition of 10 mm KCl prior to the application of 50 µm dopamine (Fig. 4*Di*,*Dii*; *n* = 7; L2 power: fast rhythm, *F*_(1,100)_ = 7.4, *p* = 0.008; slow rhythm, *F*_(1,100)_ = 33.6, *p* < 0.0001). No rhythmicity was observed at 10 mm KCl alone; however, the addition of 50 µm dopamine evoked a continuous locomotor-like rhythm with alternation in the left and right L2 and L5 and the ipsilateral L2–L5, with no transition through a multirhythmic pattern (Fig. 4*Ei*,*Eii*; L2–L2 phase: 170°; *r* = 0.91; Rayleigh test, *p* = 0.0009; L5–L5 phase: 157°; *r* = 0.72; Rayleigh test, *p* = 0.02; iL2–L5 phase: 146°; *r* = 0.95; *p* = 0.0003). This reverted back to a multirhythm with a wash in of 50 µm dopamine with regular (4 mm KCl) aCSF with no significant difference in fast or slow rhythm power under the wash conditions compared with the expected basal rhythm power (Fig. 4*Di*,*Dii*; *n* = 5; L2 power: fast rhythm, *F*_(1,92)_ = 1.3, *p* = 0.25; slow rhythm, *F*_(1,92)_ = 0.78, *p* = 0.4). These data indicate that there were no significant order effects due to sequential administration of KCl or second-messenger desensitization effects with time.

**Figure 5.**
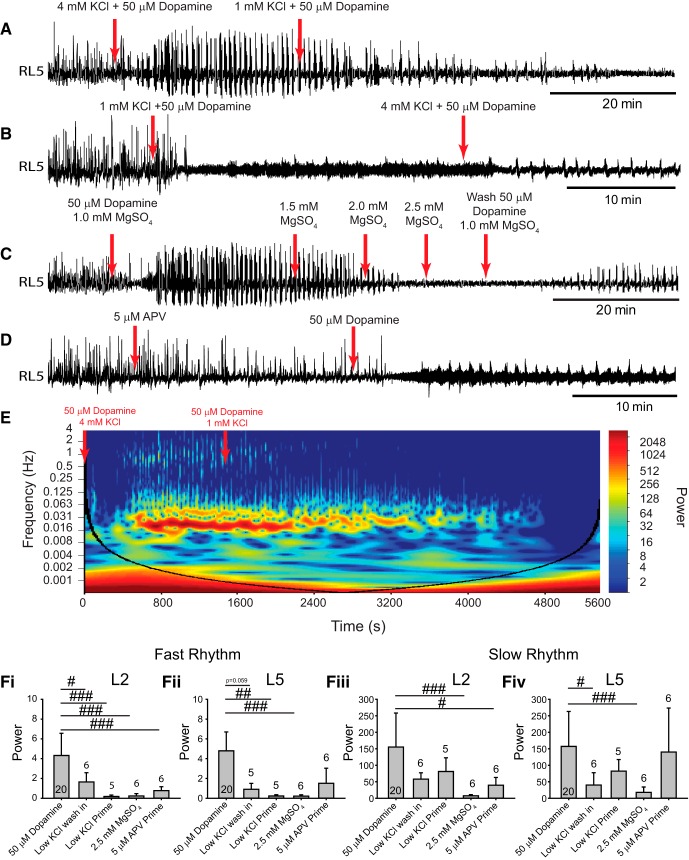
Decreasing network excitation disrupts the ability of dopamine to evoke rhythmicity. ***A–D***, Neurograms from single ventral roots within the L5 illustrate the overall effect of reducing network excitation via several pharmacological approaches on dopamine-evoked rhythmicity. ***A***, In the first experiment, network excitability was reduced by washing in aCSF containing 1 mm KCl after evoking a rhythm. ***B***, In a second experiment, preparations were recovered in low KCl (1 mm) aCSF for 1 h prior to dopamine application. ***C***, In a third experiment, excitability was reduced by sequentially increasing bath MgSO_4_ concentration (1.0–2.5 mm) after evoking a rhythm with dopamine. ***D***, In a final experiment, AP5 was bath applied to antagonize NMDA receptors (5 µm) 20 min prior to the addition of dopamine. ***E***, An example cross-wavelet frequency power spectrogram illustrates the degradation of rhythmic activity when aCSF with dopamine and low KCl washed into the bath. ***F***, Regions of interest were analyzed for cross-wavelet spectrograms between left and right L2 and L5 neurograms around fast and slow rhythm frequency bands. ***Fi***, ***Fii***, The power of the fast rhythm was reduced under all conditions in the L2 (***Fi***) and L5 (***Fii***) neurograms. ***Fiv***, Slow rhythm power was reduced in the L2s but was reduced only in the L5s by MgSO_4_ and washing in low KCl. Data are presented as the mean ± SD, with asterisks denoting a significant difference between the respective point and the rhythm at 20 min following dopamine application. Nonparametric one-way ANOVAs were performed for fast or slow rhythms in L2 and L5 power. Significant differences on *post hoc* analyses between each respective condition compared to rhythm power from 50 µm dopamine alone (#*p* < 0.05, ##*p* < 0.01, ###*p* < 0.001).

In a complimentary set of experiments, following the generation of a robust multirhythm, network excitation was reduced by washing in 50 µm dopamine in aCSF with 1 mm KCl (Fig. 5*A*). As expected, the power of the fast rhythm degraded in the L2s (Fig. 5*Fi*; *n* = 6; *p* = 0.02) and a trend toward reduced power the L5s (Fig. 5*Fii*, *n* = 5; *p* = 0.06) with no significant reduction in the slow rhythm (Fig. 5*Fiii*,*Fiv*). Similarly, 50 µm dopamine had a qualitatively smaller effect on network dynamics when it was presumably far from a transition point, as it did not evoke a fast rhythm when preparations were recovered in aCSF with 1 mm KCl for 1 h prior to dopamine application (Fig. 5*B*,*Fi*,*Fii*; L2, *p* < 0.001; L5, *p* = 0.004). In both experiments, the rhythm was slightly recovered by washing in regular aCSF (4 mm KCl; Fig. 5*A*,*B*).

**Figure 6.**
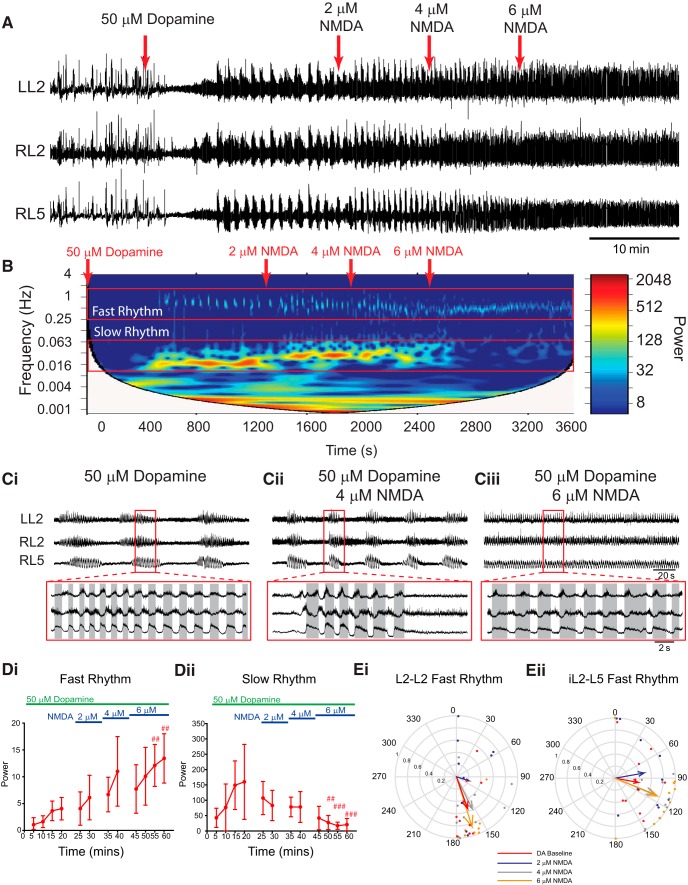
Sequential enhancement of network excitation with NMDA modulates dopamine-evoked rhythmicity. ***A***, Neurograms recorded from left and right L2 and right L5 ventral roots illustrate the experimental paradigm and resultant effect on rhythmicity. Bath application of 50 µm dopamine (DA) evoked a multirhythmic pattern of motor activity that is modulated as network excitation is boosted by sequentially increasing the concentration of NMDA in the bath (red arrows). When NMDA is increased to 6 µm, the pattern switches from multirhythmic to a single, continuous locomotor-like rhythm. ***B***, The frequency power distribution following dopamine application and subsequent excitability manipulation is illustrated in the cross-wavelet frequency power spectrogram over time with frequency on the left *y*-axis, time on the *x*-axis, and increasing power represented as warmer colors. ***C***, At 6 µm NMDA, the pattern switches from multiple rhythms to a single continuous rhythm. ***D***, Regions of interest were selected around the fast and slow rhythms, and respective frequency and power for left and right L2 and L5 neurograms were analyzed over time. Increasing network excitation increased the power of the fast rhythm and decreased the power of the slow rhythm. ***Ei***, ***Eii***, The phase and regularity of the fast rhythm is presented in the circular plots in ***Ei*** and ***Eii***, and illustrate the switch to a locomotor-like pattern at 6 µm NMDA as the vector length increases and phase moves closer to 180° in both the left and right L2s and ipsilateral L2–L5. The phase in circular plots is reported in degrees, with 180° indicating an alternating pattern and 0° being synchronous. The length of the arrows represents the mean vector length (*r*) or robustness of the pattern. Each dot represents the average phase for an individual preparation for each respective experimental condition. Data are presented as the mean ± SD, with asterisks denoting a significant difference between the respective point and the rhythm at 20 min following dopamine application (#*p* < 0.05, ##*p* < 0.01, ###*p* < 0.001) using pairwise multiple-comparisons Tukey *post hoc* analysis when significant main effects on a repeated-measures ANOVA were found.

Manipulating the extracellular KCl concentration also affects chloride concentrations. We reproduced the above experiments using NMDA to increase excitability across neuronal populations using concentrations (2–6 µm) subthreshold to those that evoke rhythmicity on their own (Fig. 6; *n* = 10). The duration of the episodes that compose the slow rhythm decreased as NMDA was increased, and differences in episode duration in L2 and L5 were no longer apparent at 2 and 4 µm NMDA (baseline: L2, 29 ± 9.5 s; L5, 22.6 ± 6.5 s; 2 µm: L2, 19.4 ± 7 s; L5, 17.2 ± 4.1 s; 4 µm: L2, 13.2 ± 4.3 s; L5, 12.6 ± 4.5 s). Boosting network excitation via NMDA decreased the frequency of the fast rhythm (L2: χ^2^_(11)_ = 64.3, *p* < 0.001; L5: *F*_(11, 99)_ = 8.8, *p* < 0.001) and increased the frequency of the slow rhythm (L2: χ^2^_(11)_ = 22.2, *p* < 0.05; L5: *F*_(11, 99)_ = 18.2, *p* < 0.001) in the L2s and the L5s. The power of the fast rhythm increased (Fig. 6*Di*; L2: X^2^_(11)_ = 82.7, *p* < 0.001; L5: *F*_(11, 99)_ = 12.1, *p* < 0.001) and the slow rhythm decreased in both the L2s and L5s (Fig. 6*Dii*; L2: χ^2^_(11)_ = 66.7, *p* < 0.001; L5: χ^2^_(11)_ = 46.6, *p* < 0.001) as excitation was increased, reflecting the rhythm switching from discontinuous multirhythm to a continuous rhythm. Likewise, the pattern of the fast rhythm became more locomotor like, as indicated by an increase in the mean vector length (*r*) pointing toward 180° (alternating) in the left and right L2s (Fig. 6*Ei*), the ipsilateral L2 and L5 (Fig. 6*Eii*), and the left and right L5s (DA baseline: L5–L5 phase: 16.1°, *r* = −0.7, *p* = 0.005; 6 µm NMDA: L5–L5 phase: 113°, *r* = 0.42, *p* = 0.2).

In an additional set of experiments, during rhythmic activity evoked by dopamine, network excitation was progressively reduced by incrementally increasing the concentration of Mg^2+^ in the bath (MgSO_4_). Increasing extracellular Mg^2+^ reduces the efficacy of NMDA-mediated synaptic transmission that is necessary for rhythmogenesis in the spinal cord (Soffe and Roberts, 1989) by impairing the removal of the voltage-dependent Mg^2+^ block in the NMDA channel. This manipulation was thus used as a means of reducing global network excitation by reducing the efficacy of excitatory synaptic transmission. A sequential increase in Mg^2+^ concentration from basal levels (1.0 mm) to 1.5 mm progressively degraded the multirhythm and fully disrupted rhythmic activity at 2.0 mm, as reflected in a significant reduction in the power of both the fast rhythm (Fig. 5*C*,*Fi*,*Fii*; *n* = 6; L2, *p* < 0.001; L5, *p* < 0.001) and the slow rhythm (Fig. 5*Fiii*,*Fiv*; *n* = 6; L2, *p* < 0.001; L5, *p* < 0.001) in both the L2s and L5s. Subsequent experiments where NMDA channels were antagonized with a low concentration of APV (5 µm) also inhibited the fast rhythm (*n* = 6, *p* < 0.001) and the slow rhythm (*n* = 6, *p* = 0.03) in the L2s but not the fast rhythm (Fig. 5*D*,*Fi*,*Fii*; *n* = 6, *p* = 0.1) or the slow rhythm (Fig. 5*D*,*Fiii*,*Fiv*; *n* = 6, *p* = 0.7) in the L5s.

### 5-HT-evoked rhythmicity is also influenced by network excitation

**Figure 7.**
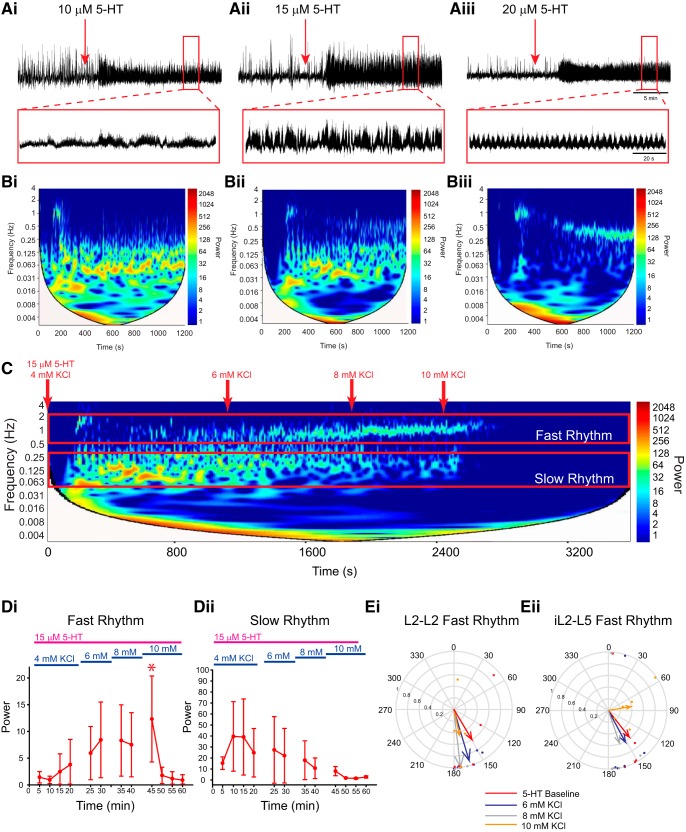
5-HT evokes multirhythmic patterns of motor activity that become locomotor like as network excitation is enhanced with KCl. ***Ai–Aiii***, Neurograms recorded in separate experiments from single L2 ventral roots illustrate that bath application of 10 µm 5-HT evokes a single slow rhythm (***Ai***), 15 µm evokes a slow and fast rhythm (***Aii***), and 20 µm evokes a single fast rhythm (***Aiii***). ***B***, Autowavelet spectrograms depicted in ***Bi–Biii*** illustrate these rhythms. ***C***, Boosting network excitation following the generation of multiple rhythms with 15 µm 5-HT caused a transition from a multirhythm to a single locomotor-like rhythm. Network manipulations are represented and highlighted as a red downward arrow in the spectrogram. ***D***, Regions of interest were selected within cross-wavelet spectrograms around the fast and slow rhythms, and the respective frequency and power for left and right L2 and L5 were analyzed over time. ***Di***, ***Div***, Boosting network excitation increased the power of the fast rhythm (***Di***) and decreased the power of the slow rhythm (***Div***). ***Ei***, ***Eii***, The bursting pattern of the fast rhythm is presented in the circular plots in ***Ei*** and ***Eii***, and illustrates an increase in the vector length at 10 mm KCl in the left and right L2s and ipsilateral L2–L5 at 8 mm KCl, but at 10 mm the length declined as activity became tonic. The phases in circular plots are reported in degrees, with 180° indicating an alternating pattern and 0° indicating a synchronous pattern. The lengths of arrows represent the mean vector length (*r*) and angle or robustness of the pattern. Each dot represents the average phase for an individual preparation for each respective experimental condition. Data are presented as the mean ± SD, with asterisks denoting a significant difference between the respective point and the rhythm at 20 min following dopamine application (*p < 0.05) from Tukey *post hoc* analysis when significant main effects on a repeated-measures ANOVA were found.

The above experiments describe how network excitation state influences the modulatory effect of dopamine on spinal motor output. Multirhythmic patterns of motor activity have been reported to be generated by a number of modulators, including noradrenaline, trace amines (Gozal et al., 2014), and 5-HT (MacLean et al., 1998; Schmidt et al., 1998). We next examined the generalizability of state dependency using 5-HT instead of dopamine. Examination of the frequency power spectrum over time following the application of low concentrations of 5-HT reveals that it too, although qualitatively different than dopamine, exhibits a slow underlying rhythm that is most apparent in the L2s and disappears as the concentration is increased (Fig. 7*A*). If this slow rhythm is a network phenomenon that is expressed at intermediate levels of excitation, then it should disappear as network excitation increases. To test this hypothesis, we sequentially increased network excitation in 2 mm KCl increments following the application of 15 µm 5-HT, which was the most effective at evoking both slow and fast rhythms simultaneously. As expected, KCl increased the robustness of the fast, locomotor-like rhythm (Fig. 7*Di*; *n* = 5; L2: *F*_(4,43)_ = 5.2, *p* < 0.001; L5: *F*_(4,44)_ = 13.9, *p* < 0.001) and decreased the power of the slow rhythm (Fig. 7*Dii*; *n* = 5; L2: *F*_(4,43)_ = 4.6, *p* < 0.001; L5: χ^2^_(11)_ = 31.1, *p* < 0.001) up to 8 mm KCl, with a 10 mm concentration causing a shift to tonic activity and a resultant decline in the power of both the fast and slow rhythms (Fig. 7*Di*,*Dii*).

## Discussion

**Figure 8.**
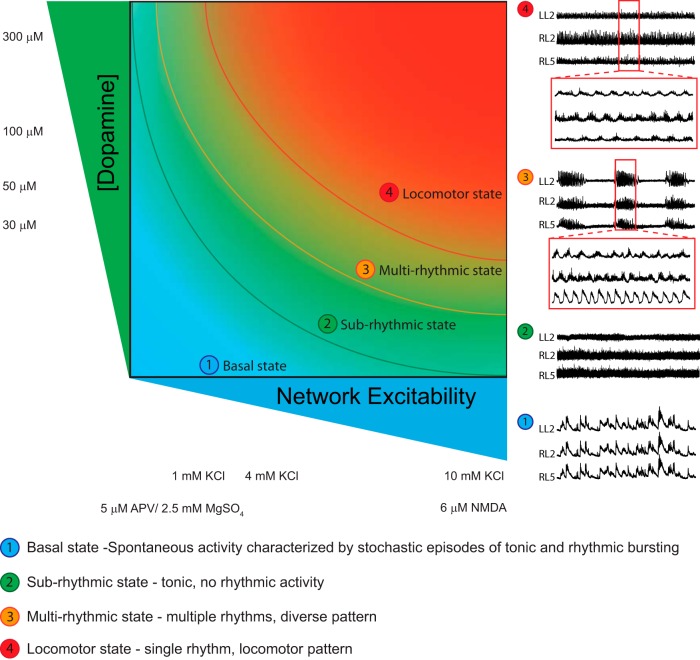
Neuromodulators evoke rhythmicity by moving the network through an excitation parameter space with the end point pattern being locomotor like. State 1 (basal state) in isolated rodent spinal cords is characterized by spontaneous network activity. Depolarization of the network moves the network into a higher excitation state (state 2), which is characterized by tonic activity with no rhythmicity. State 3 is characterized by multirhythmic patterns of motor activity where modulator-specific patterns of rhythmic motor activity may exist. Finally, in state 4, the locomotor state is characterized by continuous rhythmic activity with an alternating locomotor-like pattern expressed as a network-emergent property at the highest level of network excitation. Neurograms depicted in the schematic are from ventral root recordings in the right L5 and left and right L2 spinal segments. Curved lines in the schematic represent transition zones between network states, where dopamine would be expected to have the greatest effects on network output.

Neuromodulators play a critical role in sculpting network behavior, but these effects are state dependent (Marder et al., 2014). We made use of a well defined mouse model to explore how network excitation state dictates the modulatory effects of dopamine on spinal locomotor circuits. When we moved the excitation state of the network, we found four stable zones where dopamine produced discrete network behaviors (Fig. 8). While dopamine was used here, our work suggests that these findings are relevant to other modulators such as serotonin, and this generalizability has been pointed out in invertebrate systems (Gutierrez et al., 2013; Marder et al., 2014). Our work provides an understanding into the variability in modulatory action between preparations, which is a common challenge when studying network function *in vitro*. These results have important implications with respect to the effect of manipulations on cellular elements of the network that may move the network across state space.

**Figure 9.**
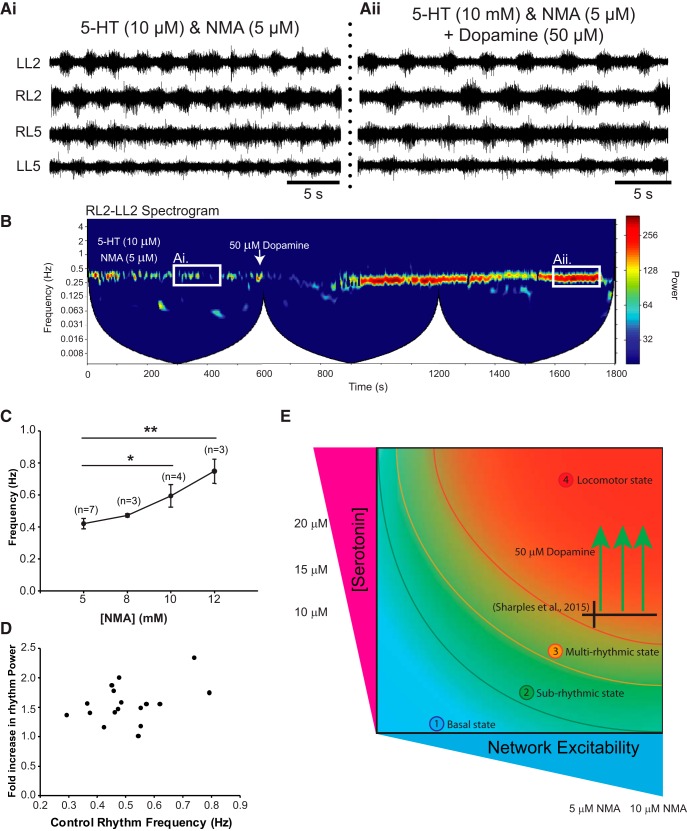
Dopaminergic modulation of state 4. Dopamine exerts a robust modulation of locomotor-like activity at a position in the state space near a transition zone; however, overall the rhythm qualitatively stays the same, and the effect does not change when network excitation is manipulated. ***Ai***, An unstable locomotor rhythm can be evoked by bath application of 10 µm 5-HT and 5 µm NMDA. ***Aii***, Dopamine reduces the frequency and stabilizes the locomotor rhythm. ***B***, This is particularly evident in the spectrogram. ***C***, ***D***, Network excitation was manipulated initially as a means of evoking rhythms of different frequencies but can also be interpreted as a network excitation state manipulation. ***D***, Regardless of the baseline frequency, the modulatory effect of dopamine on rhythm robustness (power) was the same. ***E***, The respective position of the network within the locomotor state under each modulatory and excitability condition is plotted within the state space. These data have been previously published (Sharples et al., 2015), and we provide an updated interpretation of our findings in light of our current work.

Motor networks of the lumbar spinal cord are capable of generating a diverse array of rhythmic motor patterns, including locomotion, scratching, spontaneous activity, and other movements. Most studies exploring modulation of rhythms *in vitro* focus on a continuous locomotor-like rhythms (Kiehn and Kjaerulff, 1996; Kiehn et al., 1999; Madriaga et al., 2004; Pearlstein et al., 2005; Brownstone and Wilson, 2008; Hagglund et al., 2013), which coincide with state 4 in Figure 8. While different laboratories use varying concentrations of NMDA, 5-HT, or dopamine to elicit rhythmic activity (Cazalets et al., 1992; Whelan et al., 2000; Bonnot et al., 2002; Madriaga et al., 2004; Zhong et al., 2010, 2011; Sharples et al., 2015), what is of note is that once state 4 is reached it is characterized by stable alternating bursting. Indeed, although there are subtle differences in the rhythms published by various laboratories, what is perhaps most striking are the similarities in fictive locomotor patterns. One possibility could be that subtle modulatory effects may be less apparent during a high-conductance state (Berg et al., 2007). Previous work conducted by our laboratory used an unstable locomotor rhythm generated at the lower excitation region of state 4 to reveal the stabilizing effect of dopamine (Sharples et al., 2015). Others have shown a similar effect of oxytocin (Pearson et al., 2003; Dose et al., 2014). A revised interpretation of our previous findings in light of our recent data is illustrated in Figure 9. An important caveat is that our data examine a network in a state of development. We have interpreted this based on such a network and recognize that the adult network likely responds differently and that receptor densities and functional effects are known to change during development (Perrier and Hounsgaard, 2000; Clemens et al., 2012; Keeler et al., 2016; Picton and Sillar, 2016).

### Dopamine reveals state-dependent recruitment of spinally generated rhythmicity

In the current work, the rhythmogenic effects of dopamine were dependent on the excitation state of the spinal cord. In particular, we demonstrate that manipulating network excitation can reproduce the same network output that we report only at high concentrations of dopamine. We replicated this finding using several complimentary nonspecific methods to manipulate network excitation, including increasing extracellular KCl and NMDA to increase excitation, and low KCl, Mg^2+^, and APV to reduce network excitation. Our work does not suggest that neuromodulator action is wholly dependent on network excitability as modulators have network effects beyond solely increasing network excitation. For example, we found that 5-HT can evoke rhythms that are qualitatively different from those of dopamine, but, notably, we found that the rhythms produced by 5-HT are also dependent on excitation state. Indeed, simply altering the extracellular ionic composition of potassium and calcium is sufficient to drive rhythmicity at the network and interneuronal level through the recruitment of voltage-sensitive sodium-persistent inward currents (Brocard et al., 2013).

In other states the situation is more complex. We found that the rhythm evoked by our initial concentration of dopamine that was used to modulate locomotion (Jiang et al., 1999; Whelan et al., 2000) does not resemble the rhythmic signature typically associated with walking. While the episodes of rhythmicity were regular, the phasing of the intraepisode fast rhythm between roots was less stable. Upon initial observation, one could conclude that this is the discrete rhythmic motor pattern evoked by dopamine. Others have explored rhythms that transit between states 1 and 3 (Fig. 8) and inferred a discrete modulatory function (Cazalets et al., 1992; Schmidt et al., 1998; Pearson et al., 2003; Gozal et al., 2014). It could therefore be argued that this multirhythmic pattern of activity may be a more nonphysiological motor pattern occupying state 3 and expressed under the lower levels of excitation present in reduced preparations of the nervous system.

On the other hand, neonatal rodent pups move toward the dam to suckle and toward their littermates to keep warm. While neonates do not fully bear their weight or produce functional walking movements, they do produce a diverse array of rhythmic movements of the limbs that allow them to move around the nest. Although speculative, our work could be a representation of these diverse patterns that are expressed as networks enter transition zones during development. As descending pathways mature and the excitability of spinal networks becomes more robust, stable locomotor patterns similar to state 4 would begin to emerge. Similar to developing systems, pathological rhythmic motor patterns could also emerge in adults when spinal networks move into a lower excitation state due to impaired descending activation of spinal motor networks. In other systems, such as epilepsy, altered excitation states can produce slow rhythms similar to what we have observed, but any mechanistic similarities remain speculative at this point (Jensen and Yaari, 1997).

Finally, state 1, or the basal state, in the neonatal rodent spinal cord is characterized by spontaneous activity. An interesting feature of spontaneous activity comes from previous work on the mouse where multiple rhythmic patterns were observed, from high-frequency synchronous bursts, to ipsilateral bursting and locomotor-like activity (Whelan et al., 2000). A hypothesis would then form that several transient states exist that converge onto a fourth locomotor-like state. We suspect that dopamine may be accessing a portion of these transient states and forming several identifiable and stable states. As to how dopamine can excite the cord forming regular episodes of rhythmic activity in state 3, compared with stochastic activation with spontaneous bursting activity in state 1 is unclear.

### Implications and summary

Our work illustrates at least four definable network states that restrict the effects that a neuromodulator can elicit within the lumbar spinal cord (Fig. 8). For example, the effects of dopamine on a stable locomotor-like pattern are arguably rather subtle—it does reduce the frequency and increase the amplitude of activity—but the rhythmic pattern remains qualitatively similar. This is very different if the network is transiting to state 4, where dopamine has a large impact on network function, bursting becomes much more regular, and phasing between neurograms becomes locomotor like (Sharples et al., 2015). These effects are illustrated in Figure 9. We emphasize the importance of network excitation state when studying the contributions of identifiable cell types to the production of spinally generated rhythmic activity. The removal of elements that compose the central pattern generator may move the network into lower (or higher) regions of network state space. Caution is therefore advised on inferring neuronal sub-type function based on altered patterns of motor activity before moving the network through the full range of excitation state space. This leads us to propose that an important control would be to move the network excitability in the opposite direction to the hypothesized movement of the modulator. This would allow one to detail the contribution of circuit manipulation across defined network states.

## Synthesis

Reviewing Editor: Trevor Drew, University of Montreal Decisions are customarily a result of the Reviewing Editor and the peer reviewers coming together and discussing their recommendations until a consensus is reached. When revisions are invited, a fact-based synthesis statement explaining their decision and outlining what is needed to prepare a revision will be listed below. The following reviewer(s) agreed to reveal their identity: Rune Berg.

1.line 99 contains an incomplete sentence.

2. line 247: what does the “:2” refer to?
